# Epidemiological and clinical factors of breast cancer in a population of mostly Afro-descendant women

**DOI:** 10.1590/1414-431X2025e14749

**Published:** 2025-09-12

**Authors:** J.C.B. Neiva, M.D.B. Fernandes, M.B.P. Toralles, R.S. Andrade, K.J. Erfani, M.E.F. Maia, J.M. Santana, I.M.M. Silva, J.B.L. Reis, S.O.T. Klein

**Affiliations:** 1 Centro de Ciências da Saúde, Complexo Multidisciplinar de Estudos e Pesquisas em Saúde, Universidade Federal do Recôncavo da Bahia, Santo Antônio de Jesus, BA, Brasil; 2 Programa de Pós-Graduação em Processos Interativos dos Órgãos e Sistemas, Instituto de Ciências da Saúde, Universidade Federal da Bahia, Salvador, BA, Brasil; 3 Hospital Luiz Argôlo, Santa Casa de Misericórdia, Santo Antônio de Jesus, BA, Brasil; 4 Department of Biology, Georgia State University, Atlanta, GA, USA; 5 Faculdade de Medicina, Centro Universitário de Excelência Unex, Feira de Santana, BA, Brasil

**Keywords:** Afro-descendant, Epidemiology, Inflammatory breast neoplasms, Triple-negative tumor

## Abstract

Breast cancer is the most frequent neoplasm and has the highest mortality rate among women. In the Afro-descendant population, these tumors may appear earlier and assume a more aggressive behavior. This study aimed to assess the epidemiological and clinical behavior of breast cancer in a predominantly Afro-descendant population, identify risk and prognostic factors, and compare them with already available data. Clinical and sociodemographic data were obtained from medical records and interviews with the patients involved. The variables ethnicity, age, number of children, monthly income, and education were used to describe the epidemiological profile and the results of clinical evaluation and pathological anatomy study. The immunohistochemical analysis was used to correlate the clinical characteristics of the tumors and prognosis. Afro-descendant women represented 77% of the population and the mean age at diagnosis was 54.4 years. Approximately 75% had up to 2 children, 20.5% had low income, and 37.3% had a low level of education. Infiltrating ductal carcinoma was diagnosed in 91% of patients, 70.2% had a moderate degree of differentiation, luminal subtype A was the most prevalent (39%), and a higher than global average percentage had a triple negative profile (22.9%). Early stages were identified in 53.4% of patients and only 4.8% were diagnosed with metastatic disease. The recurrence rate was 11.6%, and the mortality rate was 6.8%. The present study showed that unfavorable sociodemographic and clinical aspects, such as the high prevalence of triple-negative tumors, were not associated with a worse prognosis.

## Introduction

Currently, breast cancer is the most common, prevalent cancer and has the highest mortality rate among women (1). Although the fear of diagnosis is historical, the introduction of screening techniques and early diagnosis has contributed to gradually reduce this stigma.

With the routine performance of mammography, breast cancer began to be identified in increasingly younger women and at early stages, enabling effective, less aggressive treatments with a higher chance of cure (2). Earlier diagnosis in younger patients caused an initial increase in incidence, without being associated with increased mortality (3).

In recent decades, the incidence of aggressive breast cancer, especially in young patients, has increased, and early diagnosis and treatment is an essential predictor of cure. The routine use of immunohistochemistry (IHQ) from the 1980s has been used to identify several cell membrane receptors that interfere with the growth, development, and dissemination of breast cancer. Therefore, hormonal estrogen (ER) and progesterone (PR) receptors and c-erbB-2/Neu, also known as HER-2 (human epidermal growth factor receptor 2), have become essential targets in the growing therapeutic arsenal.

With these additional data came the perspective that there would be different types of breast cancer for which it would be possible to identify and apply specific treatments. Some epidemiological factors, such as age at diagnosis and ethnicity, especially Afro-descendant factors, have received special attention in recent years. Regularly, young women with breast neoplasia in the premenopausal phase and a low socioeconomic and demographic development index tend to develop more aggressive tumors, with a higher risk of recurrence, early metastasis, and death in a shorter time interval than older women. Younger women also have a lower expression of hormonal receptors and a higher frequency of HER-2 expression, suggesting the need for specific treatments (4,5). Additionally, Afro-descendant women have genetic variations that could increase the risk of developing triple-negative (TN) breast cancer by up to three times (6,7). Women of this ethnicity are also likely to be diagnosed with tumors in more advanced stages and consequently have lower survival.

With the improvement of IHQ techniques and the addition of other cell proliferation indices, such as the Ki67 proliferation signaling antigen, breast cancers began to be classified as follows: i) luminal A, when they express estrogen receptor (ER) (+), progesterone receptor (PR) (+), HER-2 (-), and have Ki67 less than 14%; ii) luminal B, when at least one of the hormone receptors (ER or RP) is negative or Ki67 is greater than 14% and HER-2 (-); iii) HER-2 overexpression, when patients present strongly positive HER-2 (+++) or moderately positive HER-2 (++), but with a positive FISH (fluorescent in situ hybridization) test; and iv) TN, when the hormonal receptors ER and PR and HER-2 are negative, regardless of the expression of the Ki67.

Relevant progress in oncology also showed that TN breast cancer, in addition to being the most aggressive characteristic phenotype (with risk of early metastases and death in the first two years after diagnosis), has a higher frequency in younger black women from more disadvantaged sociodemographic classes and with a family history of early-age breast cancer in first-degree relatives (8-10).

Given all this evidence and advances, this study proposes a demographic, epidemiological, and clinical socioeconomic evaluation of breast cancer in women treated in Santo Antônio de Jesus, Bahia, Brazil, where the population is predominantly Afro-descendant.

## Material and Methods

This was a retrospective and prospective cross-sectional study through the analysis of medical records and interviews. All women older than 18 years admitted to the Oncology and Hematology Center (ONCOCENTER) in Santo Antônio de Jesus, Bahia, Brazil, from May 2012 to January 2020 with breast cancer diagnosed by biopsy were included in this study. Data were collected from patients’ medical records and through a semi-structured questionnaire, which was applied personally during consultations or by telephone.

The clinical data of breast tumors included imaging exams for tumor staging, histopathological, and immunohistochemistry, all of which established the diagnosis, subtype, and tumor grade, as well as the presence or absence of lymph node involvement.

The primary treatment of patients was verified, whether surgical, adjuvant, or neoadjuvant chemotherapy, immunotherapy, hormone therapy, or radiotherapy (analyzed through the reports sent by radiotherapists). Targeted therapies with monoclonal antibodies and therapeutic evolution, including tumor recurrence or death, were also evaluated.

The Software Statistical Package for Sciences (SPSS) (version 14.0, IBM, USA) was used to perform a descriptive analysis (absolute and relative frequency, mean, and standard deviation) to identify the general and specific characteristics of the samples studied. Pearson's chi-squared test was used to determine correlations between the proportions of the categorical variables.

This study was approved by the Research Ethics Committee (REC) of the Bahian School of Medicine and Public Health on June 29, 2018, through CAAE: 87677018.9.0000.5544. Participants were duly informed about study objectives and development, possible risks, and benefits and signed the Free and Informed Consent Form (FICF) as determined by Resolution 466/12 of the National Human Health Council to ensure data confidentiality.

## Results

A total of 249 women participated in this study. The majority were from municipalities in the Recôncavo da Bahia territory (86.3%), with Santo Antônio de Jesus (44.6%) being the most prevalent municipality. The mean age was 54.39 years, and the highest incidence was in women over 50 years of age (55.4%). However, it is important to emphasize that women aged between 41 and 50 years had an incidence of 26.5%. Most participants reported being Afro-descendant (brown or black), corresponding to 76.7% of the population. The results in Table 1 show that most women were married/common law (58.4%), had 1-2 children (58.2%), a monthly income of up to three minimum wages (68.3%), and more than three dependents (54.2%).

**Table 1:** Descriptive characteristics of patients with breast cancer.

Variables	Frequency (n)	Percentage (%)
Marital status		
Single	53	21.3
Married/common law	145	58.4
Separated/divorced/widow	51	20.5
Number of children		
None	43	17.3
1-2	145	58.2
≥3	61	24.5
Monthly income		
Up to 3 minimum wages	170	68.3
4 or more salaries	77	30.9
Do not know	2	0.8
Number of dependents of monthly income		
1-2	114	45.8
≥3	135	54.2
Health insurance		
Yes	100	40.2
No	149	59.8
Schooling level		
Low	77	31
Average	121	38.5
High	51	20.5

Table 2 shows the comparisons of clinical variables between ethnic groups of Afro-descendants and non-Afro-descendants. The clinical profile of breast tumors of the study participants was performed with the analysis of the following characteristics: histological type and degree, immunohistochemical profile, clinical staging, lymph node involvement, angiolymphatic infiltrate, presence or absence of metastasis at diagnosis, site of metastasis identified at diagnosis, type of primary treatment, adjuvant radiotherapy or hormone therapy, recurrence, and death. It is noteworthy that only 17 patients died (6.8%), and of these, 6 (35%) were under 50 years of age, 7 (41.2%) had a TN tumor, and 10 (58.8%) had no health insurance.

**Table 2:** Association between clinical variables and descent in patients with breast cancer.

Variables	Afro-descendants n (%)	Non-Afro-descendants n (%)	P-value
Histological type			
Infiltrating ductal	174 (91.1)	52 (89.7)	
Infiltrating lobular	5 (2.6)	2 (3.4)	0.912
Ductal *in situ*	8 (4.2)	2 (3.4)	
Metaplastic	3 (1.6)	1 (1.7)	
Other	1 (0.5)	1 (1.7)	
Degree			
1	9 (4.7)	4 (6.9)	0.781
2	135 (70.7)	41 (70.7)	
3	47 (24.6)	13 (22.4)	
Immunohistochemical profile			
Luminal A	70 (36.6)	27 (46.6)	
Luminal B	48 (25.1)	12 (20.7)	0.603
Triple-negative	45 (23.6)	12 (20.7)	
HER-2 (+++)	28 (14.7)	7 (12.1)	
Clinical staging			
IA	48 (25.1)	21 (36.2)	
IB	3 (1.6)	0	
IIA	49 (25.7)	12 (20.7)	
IIB	27 (14.1)	6 (10.3)	0.430
IIIA	30 (15.7)	6 (10.3)	
IIIB	21 (11)	6 (10.3)	
IV	7 (3.7)	5 (5.6)	
Tumor *in situ*	6 (3.1)	2 (3.4)	
Lymph node involvement			
None	115 (60.2)	39 (67.2)	0.616
Up to 2 lymph nodes	34 (17.8)	8 (13.8)	
3 or more lymph nodes	42 (22)	11 (19)	
Angiolymphatic infiltrate			
Yes	69 (36.1)	30 (61.7)	0.034
No	122 (63.9)	28 (48.3)	
Site of metastasis at diagnosis			0.119
No metastasis	182 (95.3)	52 (89.7)	
Bone	5 (2.6)	5 (8.6)	
Lung and pleura	3 (1.6)	0	
Multiple locations	1 (0.5)	1 (1.7)	
Primary treatment			0.184
Quadrantectomy	98 (51.3)	31 (53.4)	
Mastectomy	34 (17.8)	14 (24.1)	
Neoadjuvant chemotherapy	53 (27.7)	10 (17.2)	
Palliative treatment	3 (1.6)	3 (5.2)	
Neoadjuvant hormone therapy	3 (1.6)	0	
Radiotherapy			0.234
Did not perform	19 (9.9)	10 (17.2)	
Performed without fossa	115 (60.2)	35 (60.2)	
Held with fossa	57 (29.8)	13 (22.4)	
Hormone therapy (HTx)			0.476
Did not perform	81 (42.4)	24 (41.3)	
Tamoxifen	63 (33)	20 (34.4)	
Aromatase inhibitor	35 (18.3)	13 (22.4)	
Aromatase Inhibitor + LHRL analogue	13 (0.07)	1 (0.02)	
Relapse			0.807
Yes	24 (12.6)	8 (13.8)	
No	167 (87.4)	50 (86.2)	
Death			0.981
Yes	13 (6.8)	4 (6.9)	
No	178 (93.2)	54 (93.1)	

Chi-squared test. LHRL: luteinizing hormone-releasing hormone.

The results showed a higher prevalence of infiltrating ductal tumors in both ethnic groups, with 56.6% early stage tumors at diagnosis (IA, IB, IIA, and *in situ* tumor), while approximately 38.5% had locally advanced tumors (IIB, IIIA, and IIIB). Only 4.9% of tumors had metastatic disease at diagnosis, with bones being the primary site.

Most tumors had moderate cell differentiation. Based on the IHQ profile, there was a higher prevalence of luminal tumor A, both in the Afro-descendant and non-Afro-descendant population, and in all age groups, except between 51 and 60 years, where angiolymphatic infiltrate was significantly less frequent (P=0.034).

Table 3 shows that staging was significantly associated with recurrence rate (P=0.027) and IHQ profile (P=0.026). Recurrence was recorded as “does not apply” for metastatic tumors at diagnosis. The results also showed that age and number of children were not associated with recurrence rate.

**Table 3:** Association between clinical features, pathological staging, and immunohistochemical profile with recurrence in breast cancer patients.

Variables	No relapse n (%)	Relapse n (%)	P-value
Pathological staging			
IA	65 (26.1)	4 (1.6)	
IB	2 (0.8)	1 (0.4)	
IIA	56 (22.5)	5 (2.0)	
IIB	58 (11.2)	5 (2.0)	0.027
IIIA	31 (12.4)	5 (2.0)	
IIIB	18 (7.2)	9 (3.6)	
IV	10 (4.0)	2 (0.8)	
Tumor *in situ*	7 (2.8)	1 (0.4)	
IHQ Profile			
Luminal A	91 (35.6)	6 (2.4)	
Luminal B	48 (19.3)	12 (4.8)	0.026
Triple negative	46 (18.5)	11 (4.4)	
HER-2 (+++)	32 (12.9)	3 (1.2)	

Chi-squared test. IHQ: immunohistochemistry.

**Figure 1:**
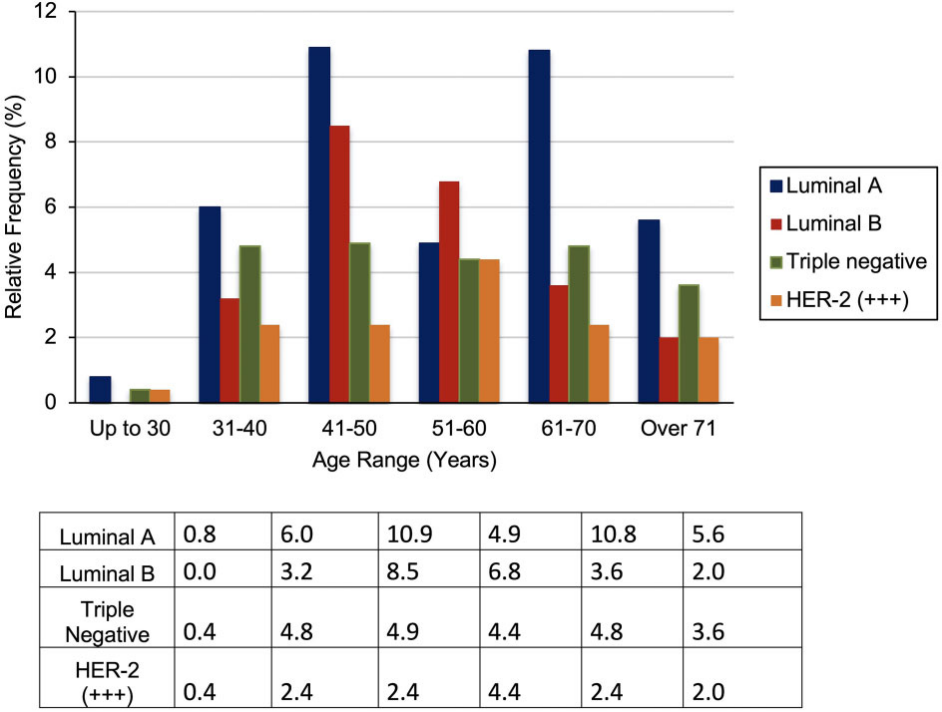
Relative frequency (%) of the immunohistochemical profile of breast tumors by age group.

HER-2 tumors (+++) were the least frequent overall, but the most frequent in the age group between 51 and 60 years. In general, the luminal type B and TN tumors had similar distributions (24.1 and 22.9%, respectively). Nevertheless, luminal B was more frequent in women between 41 and 60 years, while TN showed a similar incidence in all age groups analyzed except those under 30 years of age (Figure 1).

At diagnosis, more aggressive tumors, such as HER-2 (+++) or TN tumors, exhibited higher histological degrees. For HER-2 (+++), 62.8% (22/35) were grade II and 31% (11/35) grade III. For TN, 33% (19/57) were identified as grade II and 62% (35/57) as grade III.

Regarding primary treatment, 71.1% of patients underwent surgery, and of these, approximately 52% underwent conservative surgery (quadrantectomy). Neoadjuvant chemotherapy was the initial treatment modality in 25.3% of patients overall and in 45.6% of the patients with TN tumors.

**Figure 2:**
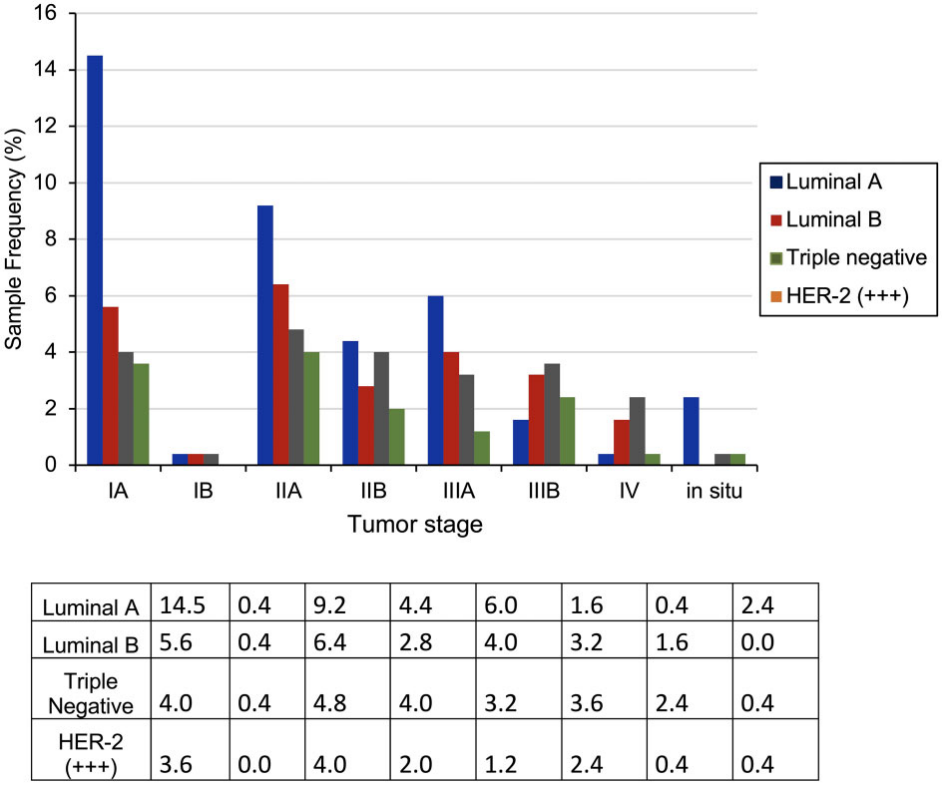
Relative frequency (%) of clinical staging and immunohistochemical profile of breast tumors.

A prevalence of 26.5% of luminal A was observed in patients diagnosed in early staging (IA, IB, IIA, and *in situ*). However, for locally advanced and advanced staging (IIB, IIIA, IIIB, and IV), a high prevalence (13.2%) of the TN profile was observed, even higher than that of luminal A (12.4%). Of the 57 cases of TN tumors, 29.8% were stage III (IIIA and IIIB), while 10.5% were stage IV (Figure 2).

Because the study population was primarily Afro-descendant and this ethnicity has a higher incidence of aggressive tumors, characteristics of TN tumors were analyzed separately. Of the 57 patients with a TN profile, 78.9% were Afro-descendants, 77.2% had an income of less than 3 minimum wages, 47.3% had not completed a high school education, and only 15.8% had completed higher education. Furthermore, 73.7% had up to 2 children, and only 26.3% had 3 children or more. Regarding histopathological characteristics, 61.4% showed histological grade 3, and 42.1% had angiolymphatic infiltrate. At diagnosis, 56.1% did not present lymph node involvement, and 17.4% had more than 4 compromised lymph nodes. During the follow-up period of these patients, 15.8% had a recurrence, and 12.3% died.

## Discussion

This study was carried out with women from the Bahian Recôncavo, where the population is predominantly Afro-descendants. Recent evidence suggests that breast neoplasia is one of the leading causes of cancer-related death in women aged 45 years (11).

Breast cancer is a highly heterogeneous pathology with complex biological characteristics, some of which are still unexplored and potentially aggressive. Management strategies, recommendations, and screening options need to consider different variables, such as age, sociodemographic factors, life habits, family history, and tumor biology.

DeSantis et al. (12) reported that the average age at diagnosis of breast cancer is 61 years in general, being 58 years in Afro-descendant women, and 62 years in non-Afro-descendant women. The present study demonstrated an average age of 54.4 years at diagnosis, 53.5 years in Afro-descendant women, and 57.3 years in non-Afro-descendant women.

Most previous studies have shown that the incidence of breast cancer is higher in women over the age of 50 (13-15), a period in which breast cancer screening tests are performed in Brazil. However, reference societies in oncology, such as the National Comprehensive Cancer Network (NCCN), the Brazilian Society of Clinical Oncology (SBOC), and the Brazilian Society of Mastology (SBM) recommend screening tests from the age of 40 to diagnose the most aggressive diseases, especially in women with greater vulnerability, such as Afro-descendants. Although the Brazilian population has extensive ethnic miscegenation and a high percentage of Afro-descendants, the Ministry of Health does not recommend mammography screening in women under 50 (15). Although the mean age at diagnosis was above 50 years, a significant portion (44.6%) of the participants in this study were younger than 50 years. In agreement with Boyle et al. (16), failure to detect early the pathology in these young women, who are Afro-descendants, socially vulnerable, and subject to a more aggressive variant, would lead to more advanced diagnoses, for which conservative treatments with minimum sequelae, secondary to chemotherapy and radiotherapy, is not possible. Consequently, the mortality rate and treatment costs could remain high. Therefore, lowering the age of mammography in breast cancer screening to 40 years could significantly increase the diagnosis of early-stage breast cancer, which would reduce mortality, especially in a socially vulnerable population subject to more aggressive variants.

Previous studies have shown that the number of pregnancies and children could be directly associated with an increased risk of TN breast cancer and a reduction in luminal subtypes. On the other hand, breastfeeding time is related to lower risk of TN tumors (17-19). According to Palmer et al. (17), more aggressive breast cancer, such as TN, mainly affects Afro-descendant women in underprivileged social conditions, with a higher number of children at an early age and a shorter breastfeeding period compared to white women. However, the present study did not find a significant association between number of children and ethnicity in the TN cancer profile.

Recently, a meta-analysis that evaluated 25 studies suggested that sociodemographic conditions, such as income and schooling, could be associated with breast cancer survival rates (20). Although the population of this study exhibited unfavorable sociodemographic characteristics, most diagnosed tumors showed low aggressive behavior, with more than 50% undergoing conservative surgery as primary treatment and very low recurrence and mortality rates. Moreover, the relationship between the recurrence rate and angiolymphatic infiltrate was significant and in favor of a low aggressive disease profile.

The results of this study corroborated recent findings regarding the high prevalence of luminal tumors A and B and the low prevalence of HER-2 (+++). However, the prevalence of TN tumor was higher (23.6% for Afro-descendant women and 20.7% for non-Afro-descendants) than in some studies, which describe 12 and 15%, regardless of ethnicity (20,21). The relationship between recurrence rate and staging at diagnosis and histological subtypes also showed statistical significance favorable to a low aggressive disease profile.

In contrast to the results reported by Li et al. (22) who showed a high incidence of tumors with grade I histological differentiation, this study found 70.7% of the tumors histologically characterized as grade II, which is a moderate degree of differentiation. Although more aggressive, 68% of the patients with grade II tumors were diagnosed at initial stages, demonstrating a more indolent tumor behavior. The most aggressive tumors, such as HER-2 (+++) and triple-negative tumors, had a higher histological degree (grades II and III), corroborating previous studies (14,23). The results showed low incidences for stages III and IV and lower risk of recurrence and early mortality compared to previous studies (24,25).

In the last decade, neoadjuvant chemotherapy has been preferably indicated because it replaces the indication of mastectomy for conservative surgery in up to 25% of cases because it allows observing the tumor's sensitivity to treatment in real-time and because it increases overall survival in patients who achieved complete pathological response (26,27). Despite this evidence, the current study did not find differences between the results obtained for patients undergoing neoadjuvant chemotherapy and those treated with surgery as the first therapeutic option. These findings are possibly associated with the high rate of early staging in the population studied, a situation in which neoadjuvant chemotherapy does not have as much impact on prognosis (28).

Regardless of the immunohistochemical profile, treatment with adjuvant radiotherapy is mandatory when conservative surgery or mastectomy is performed with high-risk disease (inflammatory breast cancer, more than 3 compromised axillary lymph nodes, compromised surgical margins, or tumors greater than 5.0 cm). In patients with TN tumors, even with localized disease submitted to mastectomy, although the indication of radiotherapy is controversial, there is a tendency to indicate it because it reduces the rate of local recurrence (29,30). The results showed that the vast majority (92%) of patients with TN breast cancer were submitted to radiotherapy, regardless of the surgery performed, corroborating the current therapeutic indications (31).

The present study offers indications that breast cancer screening could be performed at an earlier age and may increase the diagnosis of tumors at earlier stages, with better chances of less aggressive treatments and higher chances of cure, especially in the Afro-descendant population. The tumor characteristics of the population studied, such as the high prevalence of tumors with a moderate histological differentiation, high prevalence of lymph node involvement even in early disease, and high prevalence of TN tumors, were not related to a higher frequency of recurrence or mortality.

The population of this study had a high prevalence of TN tumors, but without a worse prognosis, as expected. As the results found in this study, especially those referring to TN tumors, were different from many previous studies and to better elucidate the less aggressive biological behavior of these tumors, a more detailed study of the cell biology and an analysis of the genetic profile of this population is indicated.
